# Equilibria of complexes in the aqueous cobalt(II)–*N*-(2-hydroxybenzyl)phenylalanine system and their biological activity compared to analogous Schiff base structures

**DOI:** 10.1016/j.csbj.2023.01.035

**Published:** 2023-01-27

**Authors:** Magdalena Woźniczka, Mirosława Świątek, Manas Sutradhar, Joanna Gądek-Sobczyńska, Magdalena Chmiela, Weronika Gonciarz, Beata Pasternak, Marek Pająk

**Affiliations:** a Department of Physical and Biocoordination Chemistry, Faculty of Pharmacy, Medical University of Lodz, Muszyńskiego 1, 90-151 Lodz, Poland; b Faculdade de Engenharia, Universidade Lusófona de Humanidades e Tecnologias, Campo Grande 376, Lisboa 1749-024, Portugal; c Centro de Química Estrutural, Instituto Superior Técnico, Universidade de Lisboa, Av. Rovisco Pais, 1049-001 Lisboa, Portugal; d Department of Immunology and Infectious Biology, Institute of Microbiology, Biotechnology and Immunology, Faculty of Biology and Environmental Protection, University of Lodz, Banacha 12/16, 90-237 Lodz, Poland; e Department of Organic Chemistry, Faculty of Chemistry, University of Lodz, Tamka 12, 91-403 Lodz, Poland

**Keywords:** Cobalt(II) complex, Schiff base, Stability constant, UV/Vis spectroscopy, Antimicrobial activity, Anticancer activity

## Abstract

Due to their excellent prospects in biological applications, Schiff bases and their complexes are a source of continuing interest. The present study examines the formation of four cobalt(II) complexes with the reduced Schiff base *N*-(2-hydroxybenzyl)phenylalanine (PhAlaSal) in alkaline aqueous solution by pH-metry. UV–Vis and ESI–MS studies confirmed the model of proposed species. Kinetic analysis indicated that the single- and bi-ligand cobalt(II) complexes transitioned from octahedral to tetrahedral structures. The Schiff base and its complexes detected under physiological pH were tested for antimicrobial abilities and compared with analogous structures of the Schiff base derivative, *N*-(2-hydroxybenzyl)alanine (AlaSal). The ability of these structures to influence cell growth was tested on L929 mouse fibroblasts and on cervix and gastric adenocarcinoma cancer cell lines. *N*-(2-hydroxybenzyl)phenylalanine demonstrates greater antimicrobial efficacy than *N*-(2-hydroxybenzyl)alanine but also higher cytotoxicity; however, it is nonetheless effective against cancer cells. In turn, AlaSal demonstrates low cytotoxicity for fibroblasts and high cytotoxicity for gastric adenocarcinoma epithelial cells at bacteriostatic concentration for *Helicobacter pylori* and *Candida* strains. The presence of these microorganisms in the gastric milieu supports the development of gastritis and gastric cancer; AlaSal therapy may be simultaneously effective against both. Due to their cytotoxicity, Schiff base complexes are not suitable for use against fungal and bacterial infections, but may effectively prevent cancer cell growth.

**Data availability:**

Data will be made available on request.

## Highlights

• Equilibria in the cobalt(II)–Schiff base system were evaluated.
• The speciation model was confirmed by potentiometric and spectroscopic methods.
• Kinetic analysis showed a structural change of complexes from octahedral to tetrahedral.
• Antimicrobial activity of Schiff bases and their Co(II) complexes was evaluated.
• The ability of structures to inhibit the growth of cervix and gastric adenocarcinoma cells was tested.

## Graphical Abstract

**Figure:**
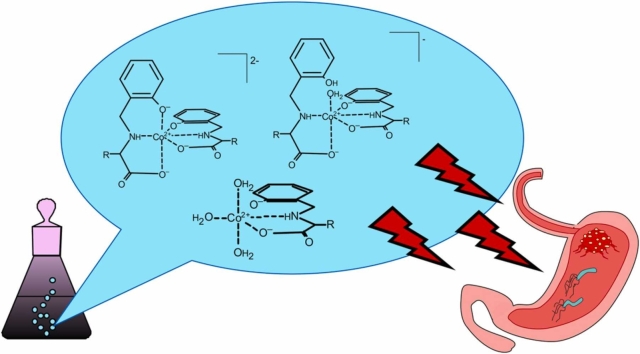

## 1 Introduction

The study of chemical equilibria provides an insight into the nature of aqueous systems by understanding the dynamics taking place in solutions [1]. Equilibrium systems provide an insight into the types of complexes formed depending on the pH of the environment, which will enable the identification of structures with potential biological properties. This knowledge can be used for synthesizing individual structures detected in solutions, and for designing new chemical compounds with medical and pharmaceutical applications. The biological properties of ligands and metal ions largely depend on the complexation process [2], [3], [4]. Although some resulting structures can cause pathological changes, others containing transition metals (iron, copper, zinc or cobalt) can be used for treatments [5], [6], [7].

There is a growing need for drugs with high efficacy and low side effects. This demand can be met by coordination chemistry, a very promising field of chemotherapy which can be used to increase the antiproliferative effect of complexes through the careful selection of metals and ligands [8], [9].

The trace element cobalt(II) is a key component of cobalamin, which plays an important role in the metabolism. It is also found in various cobalt proteins, such as methionine aminopeptidase 2, an enzyme found in humans and other mammals, and nitrile hydratase, an enzyme in bacteria that metabolizes nitriles [10]. It can therefore be assumed that complexes based around cobalt will be safer treatments for cancer or bacterial infections then existing ones [9]. Due to the good therapeutic efficacy of cobalt complexes against liver cancer cells (SK-HEP-1 and HepG2), these compounds can be used in target-based anticancer therapy [11], [12]. Also, Gowdhami et al. indicate that cobalt–Schiff base complexes may be effective chemotherapeutic drugs for cancer [13]. These complexes are capable of inducing apoptosis in breast (MCF-7) and lung (A549) cancer cells, leading to loss of cell membrane integrity, DNA damage, alteration in mitochondrial membrane potential, translocation of phosphatidylserine, induction of oxidative stress, and modulation of the expression of apoptotic pathway genes.

**Scheme 1:**
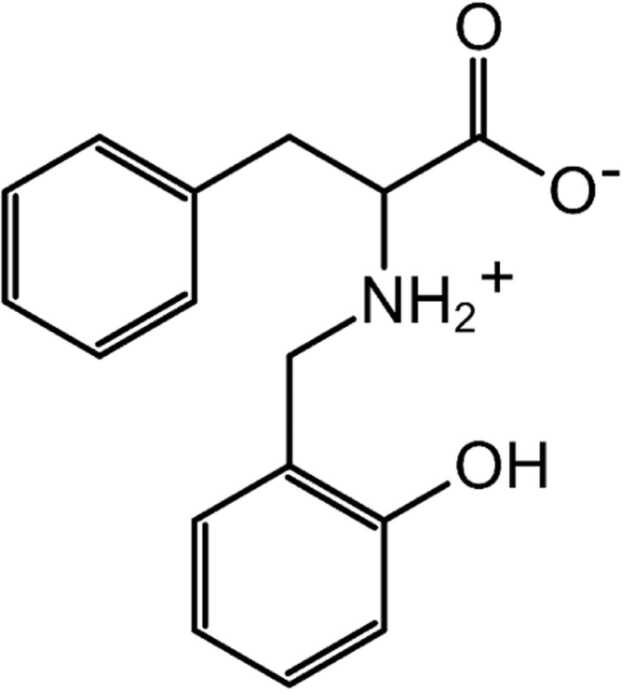
The *N*-(2-hydroxybenzyl)phenylalanine (PhAlaSal) structure as zwitter-ionic form [LH_2_].

The present study examines the complexation equilibria of cobalt(II) with the reduced Schiff base *N*-(2-hydroxybenzyl)phenylalanine (PhAlaSal, Scheme 1) in aqueous solution. PhAlaSal is formed by a synthesis reaction of salicylaldehyde with phenylalanine, an amino acid with a non-polar side chain [14]. The cobalt(II) complexes show stability in an aqueous environment, which is important for testing equilibrium systems [7]. Schiff bases, on the other hand, demonstrate high complexation potential with metal ions due to the presence of phenolic, amino and carboxyl groups that support the development of chelating compounds [8], [15], [16], [17]. Schiff base ligands and their transition metal complexes have a variety of applications as antibacterial, antifungal, antitumor, anti-HIV, anti-inflammatory and antiviral agents [18], [19], [20], [21], [22], [23], [24].

Interest in new metal-based drugs has recently grown, as complexes with Schiff base ligands have been found to accelerate gene mutations by binding to and cleaving DNA [25], [26], [27], [28], [29]. As indicated by Shebl et al., the Schiff base 1-(5-(1-(2-aminophenylimino)ethyl)− 2,4-dihydroxyphenyl)ethanone shows higher antioxidant activity than complexes [30]. The Schiff base and its complexes with cerium(III) and thorium(IV) ions also exhibit specific optical properties [31]. In addition, thanks to their high stability and reaction rate in aqueous solution and rigid structure, systems with Schiff bases demonstrate greater luminescence [32].

The search for new biological functions of Schiff base complexes has sparked interest in the cobalt(II)–PhAlaSal system, and studies have compared its properties with compounds containing the analogous Schiff base, N-(2-hydroxybenzyl)alanine (AlaSal, [33]). These studies have also focussed on the antimicrobial and anti-cancer effects of the tested complexes and ligands because chronic inflammation predisposes to the development of human cancers. For example, gastric cancer is related to *Helicobacter pylori* infection [34], [35], [36], and the opportunistic fungus *Candida albicans* increases the risk of carcinogenesis and metastasis through several mechanisms, such as production of carcinogenic byproducts, triggering of inflammation and induction of Th17 response [37]. Therefore, effective treatments need to both control infections caused by bacteria and fungi and counter their carcinogenic effects.

## 2 Experimental

### 2.1 Materials

The synthesis of *N*-(2-hydroxybenzyl)phenylalanine (PhAlaSal) has been described in [14]. Solutions of cobalt(II) nitrate hexahydrate and cobalt(II) perchlorate hexahydrate (Sigma-Aldrich, Saint Louis, USA) were titrated with EDTA disodium salt in the presence of murexide. Solutions of carbonate-free 0.1 M and 1.0 M NaOH, methanol and water (HPLC-grade) were purchased from J.T. Baker. The acid solutions from Sigma–Aldrich were standardized alkalimetrically and determined by the Gran method [38]. Potassium nitrate(V) (J.T. Baker) and sodium perchlorate monohydrate (Sigma-Aldrich) solutions were used to adjust the ionic medium. Tris-HCl (Sigma-Aldrich), sodium chloride (Chempur, Piekary Śląskie, Poland) and argon of high purity (Linde, Dublin, Irlandia) were used.

### 2.2 Potentiometric measurements

Potentiometric titrations were performed at 25.0 ± 0.1 ºC and an ionic strength of 0.1 M (KNO_3_) using a Titrando 905 automatic titrator system (Metrohm, Herisau, Switzerland) with a combined microelectrode (Metrohm LL Biotrode) filled with 3 M KCl electrolyte. The experiments were conducted in aqueous solutions over which argon was passed to remove oxygen and carbon dioxide.

Solutions with various concentrations of ligand (2 ×10^−4^–5 ×10^−3^ M) in the pH range 1.8–11.0 were used to determine the protonation constants. pH-metric titrations of the cobalt(II)−PhAlaSal system were performed at ligand concentration of 5 × 10^−3^ M and ligand-to-metal molar ratios 2:1; 3:1; 5:1. The formation constants of the aqua-hydroxo complexes of cobalt(II) were determined under the same conditions. The ionic product of water p*K*_w_, included in the equilibrium model was 13.77 [39].

The overall concentration formation constants were calculated using the Hyperquad 2013 fitting procedure according to the formula: *β*_*mlh*_ = [M_*m*_L_*l*_H_*h*_]/[M]^*m*^[L]^*l*^[H]^*h*^ [40], [41]. The species distribution curves were simulated as a function of pH in HySS 2009 [42].

### 2.3 Electrospray-ionization mass spectrometry measurements

The ESI–MS and MS/MS spectra were recorded using a Varian 500-MS LC hexapole ion-trap mass spectrometer (Palo Alto, CA, USA) with an accuracy of 0.1 for *m*/*z* values. The measurements were performed for the ligand and its system with cobalt(II) in positive and negative ion modes. The test medium was a 50/50% (v/v) methanol/water mixture, providing a more stable spray and producing smaller initial droplets than water alone or a high water content solvent [43]. No background electrolyte was added.

The concentration of PhAlaSal was 5 × 10^−3^ M in both the ligand-only and the Co(II)–ligand samples. For the complex system, the ligand-metal molar ratio was equal to 2:1. All samples were adjusted to various pH values to maximize the formation of individual complexes and ionic forms of the ligand based on potentiometric data. The samples were introduced into the ESI–MS source by continuous infusion using an instrument syringe pump at a rate of 10 μL·min^−1^. The ESI-source was operated at 5.00 kV and the capillary heater was set to 350 °C. The cone voltage was within the range 40–120 V.

### 2.4 Spectrophotometric measurements

Electronic spectra were recorded on a Cary 50 Bio spectrophotometer (Palo Alto, CA, USA) connected via a fiber optic device with a Molspin automatic titration system with a combined polymer microelectrode InLab Semi-Micro (Mettler Toledo, Greifensee, Switzerland). This made it possible to perform spectrophotometric measurements while controlling the pH value. The application of the combined polymer microelectrode enabled spectrophotometric studies in a perchlorate environment, as the nitrate ions used in the potentiometric part show significant absorption in the 300 nm region (*ε* ≈ 8), attributed to the n → π* transition [44]. Thus, pH and ionic strength (*I* = 1.0 M) were adjusted by HClO_4_ and NaClO_4_, respectively. The presence of argon in the closed thermostated vessel provided an anaerobic environment free of carbon dioxide. A constant temperature of 25.0 ± 0.1 °C was maintained during all measurements. After each addition of the sodium hydroxide solution and a suitable time delay to equilibrate the system, the pH and EMF were measured and the spectrum recorded at the selected pH values. UV absorption spectra of the ligand alone were recorded at the wavelength range 220–360 nm (total concentration 2 ×10^−4^ M). The Co(II)–PhAlaSal system was prepared in the ligand-to-metal molar ratio of 2:1, at total ligand concentration 2 × 10^−3^ M. The studies were carried out within the wavelength range 250–850 nm. The molar absorption coefficients of each species were calculated after deconvolution using HypSpec software [40].

The kinetics of the structural changes of the Co(II)–PhAlaSal complexes were studied at 25.0 ± 0.1 °C by UV–Vis absorption spectroscopy coupled to a Titrando 905 automatic titrator system and an InLab Semi-Micro combined polymer microelectrode. Measurements were taken for a Co(II)–PhAlaSal solution, prepared in an aqueous 5 mM Tris-HCl/NaCl buffer at a ligand concentration of 2 × 10^–3^ M and a ligand-metal molar ratio of 2:1. The tested solution was adjusted to a pH of about 7.5 (with NaOH). Monitoring the pH values, UV/Vis spectra were recorded under anaerobic conditions (in the presence of argon) at successive time intervals in the range of 250–850 nm.

### 2.5 Biological assay

The following solutions were prepared for the biological tests: PhAlaSal ligand and the Co(II)–PhAlaSal system at a molar ligand-metal ratio 2:1. All tests were performed on freshly-prepared solutions, adjusted to pH 7.5, and then two weeks after preparation. The solvent was 5 mM Tris-HCl/NaCl aqueous buffer. The initial concentrations of the PhAlaSal and the sum of the concentrations of all the complexes in the Co(II)–PhAlaSal system were 7.30 mM and 1.82 mM, respectively. Above complex concentration of 1.82 mM, precipitation is formed.

The total concentration of the complexes was simulated using HySS 2009. The ligand and complexes were then sequentially diluted to final concentrations of 0.06 and 0.03 mM, respectively.

To compare the metabolic activity of cervix adenocarcinoma (HeLa) and gastric adenocarcinoma (AGS) epithelial cells, assays were performed for both PhAlaSal and the analogous Schiff base *N*-(2-hydroxybenzyl)alanine (AlaSal) and their cobalt(II) complexes [33]. The solutions containing AlaSal were prepared under the same conditions as PhAlaSal and Co(II)–PhAlaSal complexes, but starting at a higher concentration (7.30 mM).

#### 2.5.1 Investigation of antimicrobial properties

The following reference bacterial strains were obtained from the American Type Culture Collection (ATCC, Manassas, USA): Gram-positive *Staphylococcus aureus* ATCC 6538 and ATCC 29213, *Staphylococcus epidermidis* ATCC 12228 and *Enterococcus faecalis* ATCC 29212, and Gram-negative *Pseudomonas aeruginosa* ATCC 27853, *Escherichia coli* ATCC 25922, *Helicobacter pylori* CCUG 17874 and ATCC 700392; in addition, three fungal strains were obtained: *Candida albicans* ATCC 10231, *Candida glabrata* ATCC 2001, and *Candida parapsilosis* ATCC 22019. One strain of *H. pylori* CCUG 17874 was from the Culture Collection, University of Gothenburg (CCUG), Gothenburg, Sweden.

Antibacterial or antifungal activities were determined using broth microdilution assay according to European Committee on Antimicrobial Susceptibility (EUCAST) recommendations, as previously described [44]. The antimicrobial activity of the formulations was assessed on the basis of minimal inhibitory concentrations (MIC), minimal bactericidal concentrations (MBC) and minimal fungicidal concentrations (MFC). MIC was determined as the lowest concentration resulting in total growth inhibition. MBC/MFC were determined by taking 10 μL of the culture from each well where no visible growth was recorded, and plating it onto the surface of Columbia Agar, supplemented with 7% (v/v) sheep's blood (*H. pylori)*, MH agar (other bacteria) or Sabouraud agar (fungi). The bacterial cultures were incubated for 24 h at 37 °C, and *Candida* for 48 h at 37 °C; a lack of microbial growth indicated bactericidal or fungicidal activity. Specifically, plates with *H. pylori* were incubated under microaerophilic conditions at 37 °C for three days. The above-mentioned tests were performed in three independent experiments. Amphotericin B, gentamicin and amoxicillin were used as standard antimicrobials.

#### 2.5.2 In vitro cell culture

The mouse fibroblasts L929 (LGC Standards, Middlesex, UK), human cervix adenocarcinoma epithelial cells HeLa (CCL-2, ATCC, Manassas, USA), and human gastric adenocarcinoma epithelial cells AGS (CRL-1739, ATCC, Rockville, MD) were cultivated in 25 cm^2^ tissue culture flasks in RPMI-1640 medium (Biowest, Nuaillé, France) supplemented with 10% heat-inactivated Fetal Bovine Serum - FBS (Biowest) and the antibiotics penicillin (100 U/mL) and streptomycin (100 μg/mL, Biowest) under standard conditions (37 °C, 5% CO_2_). Cell culture medium was replaced two or three times per week to keep the cells in log phase and the confluent cell monolayer was treated with a 0.25% trypsin solution to passage.

In accordance with ISO norm 10993–5 (Biological evaluation of medical devices — Part 5: Tests for in vitro cytotoxicity), in vitro cytotoxicity testing was performed using L929 mouse fibroblasts and AGS and HeLa cancer cells. The initial cell viability ranged from 93% to 95%, as confirmed by exclusion of trypan blue dye.

##### 2.5.2.1 MTT reduction assay

The viability and growth of L929, AGS and HeLa cells in the presence of tested components were estimated by MTT assay [(3-(4,5-dimethylthiazol-2-yl)− 2,5-diphenyltetrazolium bromide)] as recommended by the Food and Drug Administration (FDA) and the International Organization for Standardization (ISO) [33]. Briefly, the cells in the culture medium were seeded in 96–well plates (2 ×10^5^ cells/well) for 24 h at 37 °C, 5% CO_2_.

The tested compounds were prepared directly and stored for two weeks. They were diluted in cRPMI‐1640 medium at concentrations of 5, 2.5, 1.25, 0.5, 0.25 and 0.1 mg/mL. They were then added to the cells (100 μL/well), and the mixture was incubated under standard conditions for 24 h. Following incubation, the cell monolayers were carefully screened using light microscopy to evaluate cell morphology, as recommended by ISO norm 10993–5, (International Organization for Standardization, 2009). The level of reduced MTT in the test and control cell cultures (culture medium only, 100% cell viability) was determined spectrophotometrically at OD 570 nm after dissolving in acidified alcohol. The percentage of living cells was then determined in the cell cultures, in the milieu of tested materials. The substance was considered non-cytotoxic for cell viability values of> 70% [33].

##### 2.5.2.2 DAPI staining of cell nuclei

The nuclei of L929, AGS and HeLa cells were stained as previously described [45] using 4′,6-diamidino-2-phenylindole (DAPI), a fluorescent dye with a strong affinity to the AT base pair in DNA. After 24 h of stimulation with decreasing concentrations of the test compounds, i.e. Co(II) ions, PhAlaSal, AlaSal and complexes in the Co(II)–PhAlaSal and Co(II)–AlaSal systems, the cells were fixed with 4% formaldehyde, and stained with DAPI solution (2.5 µg·mL^−1^) for 15 min at room temperature. Preparations were viewed under a fluorescent microscope (Zeiss, Axio Scope, A1, Oberkochen, Germany), at a wavelength of 358 nm (excitation) and 461 nm (emission). The percentage of cells demonstrating a blebbing nucleus was assessed.

#### 2.5.3 Statistical analysis

Statistical significance was accepted at a *p*-value< 0.05. All comparisons were made using the Kruskal-Wallis test. Data are presented as mean values± SD. The statistical analysis was performed using STATISTICA 13 PL software (Stat Soft, Kraków, Poland).

## 3 Results and discussion

### 3.1 Protonation equilibria of the ligand

Our potentiometric studies in aqueous solution allowed us to identify three protonation constants of PhAlaSal, which are shown in Table 1. The stepwise dissociation constants p*K*_a1_, p*K*_a2_ and p*K*_a3_ correspond to the protonated carboxyl, amino and phenolic groups, respectively. The pH-dependent ionic forms of the ligand are presented in the species distribution graph (Fig. S1). Although similar constants were obtained previously in a dioxan: water environment, the authors report only two protonation constants above pH 3.5 [46]. In contrast, Ozawa et al. determined three protonation constants in aqueous solution with values similar to those shown in Table 1; this method used spectrophotometric or potentiometric measurements depending on pH [47]. The dissociation constants of PhAlaSal relating to the amino and phenolic groups are slightly lower than those observed for the reduced Schiff base, *N*-(2-hydroxybenzyl)alanine (AlaSal) [33]. The electrodonating potential of the methyl group increases the electron density on the oxygen and nitrogen atoms, resulting in more basic AlaSal donor groups and higher p*K*_a_ values. On the other hand, the presence of a benzyl group in the PhAlaSal molecule results in lower p*K*_a_ values compared to AlaSal due to its electron-withdrawing effect and increased acidity of the donor groups [41], [48].

**Table 1:** Decimal logarithms of overall protonation and formation constants in the Co(II) – PhAlaSal system, *β*_*mlh*_ = [M*m*L_*l*_H_*h*_]/[M]^*m*^[L]^*l*^[H]^*h*^ at 25.0 ± 0.1 °C, *I* = 0.1 and UV/Vis spectral data. Standard deviations, given in parentheses after overall protonation and stability constants, refer to random errors only.

Species	log_10_*β*_*mlh*_	Stepwise dissociation constants	Related constants	*λ*_max_ (*ε*_max_)
[L]^2-^				238 (8.1 × 10^3^) 291 (3.4 × 10^3^) ∼330^sh^ (5.5 × 10^2^)
[LH]^-^	9.94(3) (OH)	p*K*_a3_ 9.94		237 (6.0 × 10^3^) 292 (2.3 × 10^3^) ∼331^sh^ (5.6 × 10^2^)
[LH_2_]	18.13(6) (NH_2_^+^)	p*K*_a2_ 8.19^a^		∼242^sh^ (1.9 × 10^3^) 276 (2.2 × 10^3^) ∼298^sh^ (7.9 × 10^2^)
[LH_3_]^+^	21.03(8) (COOH)	p*K*_a1_ 2.90^b^		
*σ***;***n*^c^	8.15; 960			
[CoL]	6.41(13)			
[CoL_2_]^2-^	11.11(5)			469 (90)
[CoL_3_]^4-^	15.26(8)			
[CoL_2_H]^-^	19.60(9)		9.66^d^	
*σ*; *n*^c^	2.92; 156			

a $pK_{a2}=\log K_{\text{L}{\text{H}}_{2}}^{\text{LH}}=\log\beta_{\text{L}{\text{H}}_{2}}-\log\beta_{\text{LH}}$

b $pK_{a1}=\log K_{\text{L}{\text{H}}_{3}}^{\text{L}{\text{H}}_{2}}=\log\beta_{\text{L}{\text{H}}_{3}}-\log\beta_{\text{L}{\text{H}}_{2}}$

c *σ* – the value of the normalized sum of squared residuals, *n* – number of refined parameters.

d $\log K_{\text{Co}{\text{L}}_{2}H}^{\text{Co}}=\log\beta_{\text{Co}{\text{L}}_{2}H}-\log\beta_{\text{LH}}$

The ESI–MS spectra performed for PhAlaSal correspond to the potentiometric results. As shown in the tandem mass spectrum (Fig. S2), the adduct [LH_2_ + NO_2_ + H]^+^ (*m/z* = 318.0) (Fig. S3) fragments into several types of ions. The decomposition of the adduct contributes to the formation of the ion [LH_2_ + H]^+^ (*m/z* = 272.0). According to the fragmentation pattern, the loss of the hydroxyl and subsequent benzyl groups from [LH_2_] resulted in ions with *m/z* = 255.0 and *m/z* = 166, respectively. In the next step, two competitive fragmentations occur: loss of the amino group, resulting in an ion with *m/z* = 152.0, and detachment of -OH from the carboxyl group, forming an ion with *m/z* = 149.0.

Regarding the positive-ion spectra of PhAlaSal, the deprotonated form of [LH_2_] is observed as an adduct [LH_2_ + NO_2_ + H]^+^ (Fig. S3). In addition, an Na^+^ adduct (*m/z* = 278.0) of the fragment ion *m/z* = 255.0 was detected; alkalization of the environment increases its relative intensity, making it one of the dominant ions at pH 9.0. A signal for a fragment ion (*m/z* = 193.0) obtained by detaching the C_6_H_5_- group from [LH]^-^ was also detected.

In negative-ion mode (Fig. S4), the ligand [LH]^-^ (*m/z* = 270.0) showed a trace presence. However, the dominant form in the pH range 5.5–11.0 is a fragment ion with *m/z* = 147.0 obtained by deprotonation of ion with *m/z* = 149.0.

### 3.2 Complex formation equilibria

**Scheme 2:**
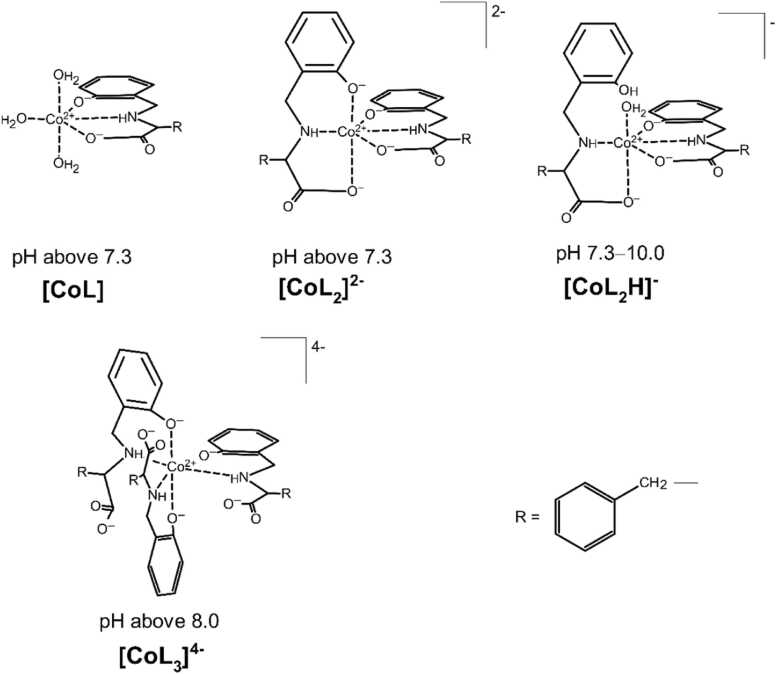
Suggested coordination modes of the complexes in the Co(II)–PhAlaSal system with regard to pH.

**Fig. 1:**
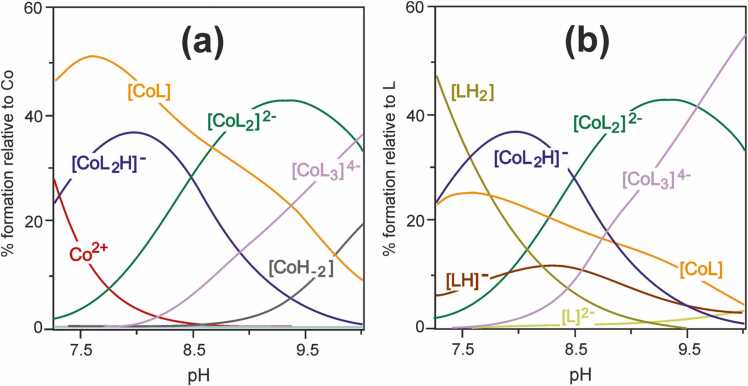
Species distribution curves as a pH function in the Co(II)–PhAlaSal system at ligand-to-metal molar ratio 2:1 relative to **(a)** Co(II), **(b)** ligand; *C*_PhAlaSal_ = 5.0 × 10^−3^ M.

Potentiometric studies carried out in a narrow pH range (approximately 7.3–10.0) confirmed the formation of four complexes in the Co(II)–PhAlaSal system (Scheme 2). Below this pH range, up to a pH of about 2.2, a white precipitation appeared in the solution, most likely connected with low water solubility of species formed in the system. The affinity of the complexes for water affects the solubility of these structures, namely, the negatively-charged forms and polar structures are the most soluble, followed by the positively-charged or neutral complexes with protonated side groups [49]. In addition, the Co(II) aqua-hydroxo complex [CoH_-2_] precipitates in a more alkaline medium, making it impossible to continue measurements. Table 1 shows the overall stability constants of the obtained complexes, and their distribution is presented in Fig. 1.

Patel et al. determined the stability constants of PhAlaSal complexes with divalent transition metals, Ni and Cu, in the dioxan: water medium [46]. Their findings are close to those given in Table 1: the order of stability constants for each interaction was found to be Co(II)<Ni(II)<Cu(II), following the Irving-Williams order for divalent ions [50]. The cobalt(II) system offers the advantage that a greater number of complex species is formed in solution.

Discrepancies can be seen between the stability constants of the cobalt(II) complexes with PhAlaSal, and with AlaSal [33], which is likely due to the presence of different side groups in the ligand molecules corresponding to the original amino acids. However, the presence of analogous coordination sites between the ligands indicates that the two systems have similar complexation modes. This is also influenced by the lack of significant side group interactions with the metal ion. X-ray analysis revealed that in the Ni(II) complex with Schiff base obtained from salicylaldehyde and L-phenylalanine, the imine, phenolic and carboxyl groups of the Schiff base ligand provide equatorial coordination with the metal ion [51]. Furthermore, the tridentate AlaSal binds to the central ion in the equatorial sites via amino, phenolic and carboxyl groups. Therefore, the most likely mode of coordination in the [CoL] complex with PhAlaSal is the formation of five-membered and six-membered chelate rings in the equatorial plane (Scheme 2). Zhao et al. showed that these two rings are not really coplanar, but the stability of the complex is enhanced [51].

In the case of the fully-deprotonated bi-ligand complex [CoL_2_]^2-^, it is likely that the nitrogen atom of the second molecule of PhAlaSal occupies the last equatorial position (Scheme 2). This type of coordination has been previously observed in both the X-ray crystal form of AlaSal with Schiff base ligands, and the aqueous solution [33], [52].

[CoL_2_H]^-^ species with a single protonated phenolic group are also observed in the pH range of [CoL] formation (Fig. 1). The [CoL_2_H]^-^ complex shows a lower related stability constant than the completely deprotonated [CoL_2_]^2-^ (Table 1). The protonated PhAlaSal molecule indicates classical amino acid chelation by the amino nitrogen and the carboxyl oxygen, most likely occurring in the equatorial and axial positions (Scheme 2). The protonated phenolic group remains non-coordinating and appears to participate in the hydrogen bonds with water, or between the amino and phenolic groups of the same molecule, as confirmed by the crystal structures of amino acid-derived ligands, such as L-tyrosine [53], [54]. In contrast, the second ligand molecule, fully deprotonated, probably coordinates in an equatorial {O^-^_phenolic_,N,O^-^_carboxyl_} chelation mode, like AlaSal in the cobalt(II) system [33]. Deprotonation of the [CoL_2_H]^-^ complex to [CoL_2_]^2-^ proceeds according to the Eq. (1):

(1) [CoL_2_H]^-^ = [CoL_2_]^2-^ + H^+^·

$pK_{\text{Co}{\text{L}}_{2}}^{\text{Co}{\text{L}}_{2}H}=8.49$is lower than deprotonation constant for the phenolic proton of the corresponding free ligand (p*K*_a3_ = 9.94). This enables the formation of the [CoL_2_]^2-^ above pH 7 (Fig. 1).

Crystal structures with an excess of deprotonated donor groups relative to coordination sites desmonstrate a distinct mode of complexation compared to single- and bi-ligand species [55]. Our findings indicate that in the [CoL_3_]^4-^ complex, the equatorial positions are occupied by one phenolic oxygen and three amino nitrogens derived from three PhAlaSal molecules (Scheme 2). Most likely, the other two phenolic groups are bound to the metal ion through the axial sites. This mode of coordination affects the different planes of the ligand molecules and thus prevents the destabilization of the complex caused by the electrostatic repulsion between negatively-charged, uncoordinated carboxyl groups [44], [46].

For the Co(II) system with PhAlaSal, ESI–MS spectra were recorded at various pH values in positive and negative ion mode, including the acidic medium before the precipitate appeared. However, as indicated by the spectra, the complexes do not yet form at pH 2.0, and the observed form is the adduct [LH_2_ + NO_2_ + H]^+^ (*m/z* = 318.0) (Fig. S5). In turn, the Na^+^ adduct with *m/z* = 278.0, containing fragment ion *m/z* = 255.0, has a higher relative intensity at three pH values (7.5, 8.2, 9.5) in positive ion mode. Negative adducts of complexes with one ligand molecule [CoL + OH]^-^ (*m/z* = 344.9) and [CoL + NO_3_]^-^ (*m/z* = 389.9), as well as the cobalt(II) aqua-ion ([Co(II) + 3NO_3_]^-^, *m/z* = 244.9) were also detected in alkaline media (Fig. S6). Confirming the potentiometric data, the bi-ligand complexes and their adducts were recorded in both ionic modes: [CoL_2_ + 4Na + H_2_O]^2+^ (*m/z* = 353.5), [CoL + fragment ion *m/z* = 255.0]^+^ (*m/z* = 582.9), [CoL_2_H + H_2_O + 2 H]^+^ (*m/z* = 617.9), [CoL_2_ + H_2_O + 3 H]^+^ (*m/z* = 617.9), [CoL_2_H + 2Na]^+^ (*m/z* = 643.9), [CoL_2_ + 2Na + H]^+^ (*m/z* = 643.9), [CoL_2_ + 3Na + 2 H_2_O]^+^ (*m/z* = 701.9), [CoL_2_H + NaOH + CH_3_OH + H_2_O]^-^ (*m/z* = 687.9), [CoL_2_ + NaOH + H + CH_3_OH + H_2_O]^-^ (*m/z* = 687.9). Furthermore, one adduct of the tri-ligand complex [CoL_3_ + Na + 5 H]^2+^ (*m/z* = 447.0) and associate species [CoL_2_ + CoL + Na + 3 H]^2+^ with *m/z* = 475.4 were detected throughout the tested alkaline pH range.

### 3.3 UV-Vis spectra

UV–Vis absorption spectra for the ligand were only scanned within 200–360 nm due to the lack of absorption above those wavelengths (Fig. S7a). A wide range of pH values was used to identify different ligand forms with characteristic electronic absorption spectra. The HypSpec deconvolution enabled calculation of the molar absorption coefficients (*ε*) for the [LH_2_], [LH]^-^ and [L]^2-^ forms (Fig. S7b, Table 1). The isosbestic points were found at 260 and 284 nm, 262 and 274 nm, which corresponds to deprotonation steps between [LH_2_] and [LH]^-^ as well as [LH]^-^ and [L]^2-^, respectively. Moreover, the occurrence of an isosbestic point at 244 nm in the acidic medium confirms the presence of the [LH_3_]^+^ form based on the existence of equilibria between [LH_3_]^+^ and [LH_2_], also observed in potentiometric studies (Fig. S1).

As indicated by the UV-Vis absorption spectra for the Co(II)–PhAlaSal system and as already observed for the potentiometric titration (Fig. 1), the complexes form above pH 7.0 (Fig. S8a). The presence of the precipitation at pH around 2.0–7.0 did not allow for the registration of spectra in this range. Moreover, in the acidic medium, only the band associated with Co(H_2_O)_6_^2+^ was observed (*ε*_max_ = 4.6 at 515 nm [56]). In the alkaline medium, the blue shift of the *d-d* bands relative to the cobalt(II) aqua-ion confirms the presence of the octahedral complex [CoL_2_]^2-^ by HypSpec deconvolution (Fig. S8b, Table 1). The occurrence of an isosbestic point (445 nm) at pH above 9.5 most likely indicates the appearance of a tri-ligand complex as an effect of the equilibrium between [CoL_2_]^2-^ and [CoL_3_]^4-^ species. In the 370–430 nm range, weak shoulders are visible, most probably assigned to the LMCT bands. This may suggest the coordination of the phenolic group and the formation of the dianionic form of the ligand, as described previously [33], [57], [58].

**Fig. 2:**
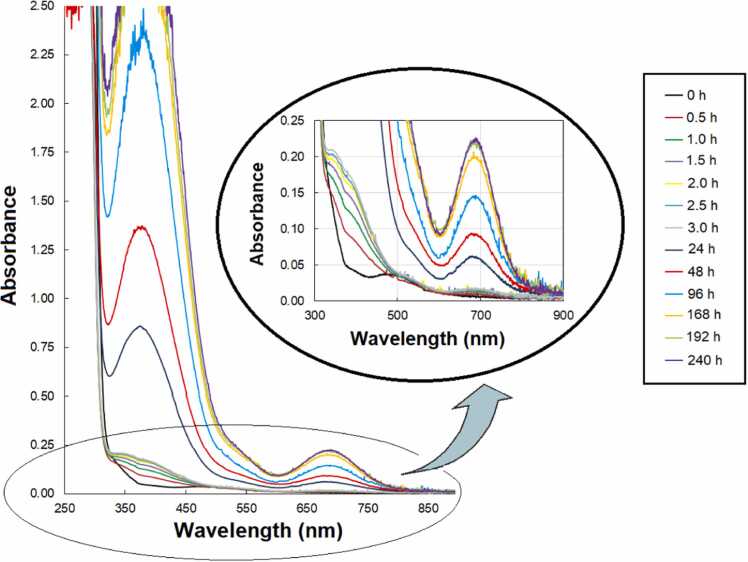
Absorption spectra of complexes in the Co(II)–PhAlaSal system (*C*_PhAlaSal_ = 2 ×10^−3^ M, at ligand-to-metal molar ratio 2:1) at pH 7.5, recorded at consecutive time intervals.

At a pH of about 7.5, which is important for biological research, a time-dependent change in absorbance was determined for the Co(II)-PhAlaSal system (Fig. 2). In the initial phase of the test, the species observed in the potentiometric measurements were identified, as confirmed by the presence of a band characteristic for octahedral cobalt(II) complexes (maximum absorbance at about 469 nm) (Fig. 1). Maintaining anaerobic conditions, within the next defined time interval, the peak was red-shifted and a new band was recorded at 680 nm, assigned to the transition ^4^A_2_ → ^4^T_1_(P) typical for tetrahedral Co(II) complexes [59], [60], [61]. In addition, a band at 380 nm appeared, probably related to the CT transition in tetrahedral complexes. Similarly to the complexes formed in the cobalt(II) system with AlaSal [33], the single- and bi-ligand Co(II)–PhAlaSal species changed their structures from octahedral to tetrahedral. A substantially rectilinear increase in absorbance at 680 nm was noted over 192 h (eight days of measurements), after which the system begins to stabilize.

### 3.4 Biological activity

The antimicrobial activity of the PhAlaSal and its cobalt(II) complexes was determined based on MIC/MBC/MFC values (Table 2). For comparison, MIC/MBC/MFC values for standard antibiotics (gentamicin, amphotericin B and amoxicillin) were also tested. To account for differences in molecular weight, the MIC/MBC/MFC values were expressed in mM to allow comparison between the antimicrobial activity of the Co(II)–PhAlaSal complexes with those of the ligand alone and the analogous Schiff base derivative, AlaSal.

**Table 2:** Antimicrobial activity of the ligand PhAlaSal and complexes in the Co(II)–PhAlaSal system, prepared directly and stored for two weeks, shown as minimal inhibitory concentration (MIC) and minimal bactericidal concentration (MBC) or minimal fungicidal concentration (MFC). Antibiotics: gentamicin, amoxicillin and amphotericin used as antibacterial and antifungal reference substances; (-) not tested.

Microorganism			MIC/MBC/MFC (mM)		MIC = MBC/MFC (mM)		
	Co (II)–PhAlaSal complexes		PhAlaSal		Gentamicin	Amphotericin B	Amoxicillin
	MIC	MBC	MIC	MBC			
Gram-negative bacteria							
*Pseudomonas aeruginosa* ATCC 27853	> 1.82	> 1.82	7.30	> 7.30	< 0.008	-	-
*Escherichia coli* ATCC 25922	> 1.82	> 1.82	7.30	> 7.30	< 0.004	-	-
*Helicobacter pylori* CCUC 17874	1.82	1.82	3.65	> 7.30	-	-	< 0.001
*Helicobacter pylori* ATCC 700392	1.82	1.82	3.65	> 7.30	-	-	< 0.001
Gram-positive bacteria							
*Enterococcus faecalis* ATCC 29212	> 1.82	> 1.82	7.30	> 7.30	< 0.26	-	-
*Staphylococcus aureus* ATCC 29213	> 1.82	> 1.82	3.65	> 7.30	< 0.002	-	-
*Staphylococcus aureus* ATCC 6538	> 1.82	> 1.82	3.65	> 7.30	< 0.002	-	-
*Staphylococcus epidermidis* ATCC 12228	> 1.82	> 1.82	3.65	> 7.30	< 0.002	-	-
Fungi							
*Candida albicans* ATTC 10231	> 1.82	> 1.82	3.65	> 7.30	-	< 0.001	-
*Candida glabrata* ATCC 2001	> 1.82	> 1.82	3.65	> 7.30	-	< 0.001	-
*Candida parapsilosis* ATCC 22019	> 1.82	> 1.82	3.65	> 7.30	-	< 0.001	-

The Co(II)–PhAlaSal complexes exhibited the highest bacteriostatic and bactericidal activity against two *H. pylori* strains, with MIC and MBC of 1.82 mM; the MIC/MBC/MCF values were> 1.82 for all other tested microorganisms (Table 2). Both octahedral (solution prepared directly) and tetrahedral (solution stored for two weeks) structures were tested, with all species having the same bacteriostatic and bactericidal activity. In turn, for PhAlaSal alone, the MIC values were equal to 3.65 mM for *H. pylori*, *S. aureus*, *S. epidermidis* as well as fungi, and 7.30 mM for *P. aeruginosa*, *E. coli* and *E. faecalis* (Table 2). For all tested microorganisms, the MBC values of the ligand were> 7.30 mM. Interestingly, PhAlaSal indicates lower MICs for the two *H. pylori* strains and most of tested Gram-positive bacteria compared to AlaSal [33]. These greater antimicrobial properties may be due to the presence of a benzyl group, in addition to the electron-donating amino nitrogen and hydroxyl oxygen of the benzene ring [62], [63].

Increasing the hydrophobic ligand shell around the cobalt(II) ion as result of charge delocalization enhances the ability of the complex to penetrate the bacterial cell membranes, limiting growth by disrupting cell respiration and blocking protein synthesis [5], [18], [64].

Thus, the coordination of the donor atoms with the metal(II) center seems to be a key factor leading to antimicrobial activity [65]; indeed, the MIC and MBC values for Co(II)–PhAlaSal complexes were found to be lower than those for the ligand alone against *H. pylori* (Table 2). Unfortunately, it was not possible to obtain accurate MIC/MBC/MFC values for other strains due to precipitation at higher complex concentrations. Also, a benzyl group in the ligand molecule contributes to the increase of the antimicrobial activity of the complexes by enhancing their lipophilic nature. Bit et al. note that the complex with 3-hydroxybenzaldehyde-DL-phenylalanine showed greater activity than other species containing an α-amino acid-derived Schiff base [18]. Interestingly, the Co(II)–PhAlaSal complexes show the same potent inhibitory activity towards *H. pylori* as free cobalt(II) [66], whose MIC is comparable to those of some antibiotics commonly used in eradication therapies for these strains [67]. In addition, the minimal bactericidal concentration of these complexes is four times lower than for the metal ions.

The MIC/MBC/MCF values of the classical antibiotics (Table 2) present significant quantitative differences in relation to the tested compounds. As in the case of other aqueous systems [33], [44], the high MIC/MBC/MCF values for the Co(II)–PhAlaSal system result from the fact that they are determined for the total concentration of all complexes present in solution at pH 7.5, i.e. [CoL], [CoL_2_H]^-^ and [CoL_2_]^2-^ (Fig. 1). In addition, the ligand [LH_2_] is one of the dominant forms at this pH, showing higher MIC/MBC values than the complexes (Table 2).

**Fig. 3:**
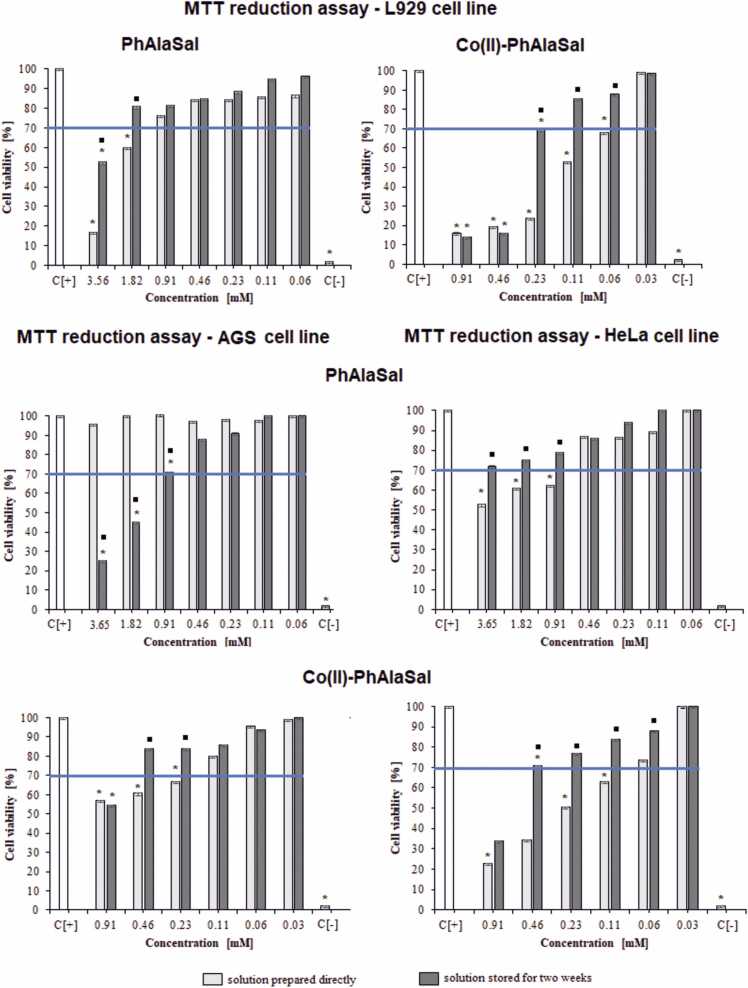
Cytotoxic effect of the studied forms: PhAlaSal, Co(II)–PhAlaSal complexes and Co(II) ions towards L929, AGS and HeLa cells. The cytotoxicity was assessed by MTT [(3-(4,5-dimethylthiazol-2-yl)− 2,5-diphenyltetrazolium bromide)] reduction assay. The cell viability was calculated for four experiments including three repeats for each compound. Complete RMPI-1640 medium (cRPMI) was used as a positive control (C+) of cell viability (100% viable cells) and 0.03% H_2_O_2_ as a negative control (C−) of cell viability (100% dead inactive cells). Statistical significance in Kruskal-Wallis test: * ■ p < 0.05; * untreated cells vs. cells treated with tested solution (solution prepared directly or solution stored for two weeks); ■ solution prepared directly vs. solution stored. The blue line indicates the minimal percentage of viable cells (70%) required to confirm the compound as non-cytotoxic at the in vitro level.

**Fig. 4:**
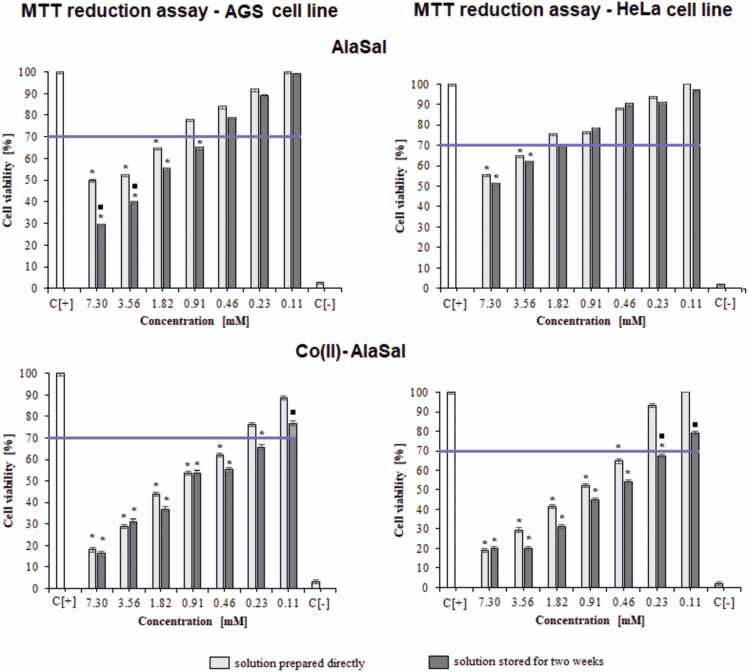
Cytotoxic effect of the studied forms: AlaSal and Co(II)–AlaSal complexes towards AGS and HeLa cells. The cytotoxicity was assessed by MTT [(3-(4,5-dimethylthiazol-2-yl)− 2,5-diphenyltetrazolium bromide)] reduction assay. The cell viability was calculated for four experiments including three repeats for each compound. Complete RMPI-1640 medium (cRPMI) was used as a positive control (C+) of cell viability (100% viable cells) and 0.03% H_2_O_2_ as a negative control (C−) of cell viability (100% dead inactive cells). Statistical significance in Kruskal-Wallis test: * ■ p < 0.05; * untreated cells vs. cells treated with tested solution (solution prepared directly or solution stored for two weeks); ■ solution prepared directly vs. solution stored. The blue line indicates the minimal percentage of viable cells (70%) required to confirm the compound as non-cytotoxic at the in vitro level.

PhAlaSal in the freshly-prepared solution significantly diminished the ability of L929 cells to reduce MTT (40–83% of dead cells) within the range 1.82–3.65 mM, whereas the ligand stored for two weeks showed a significant inhibitory effect only at a concentration of 3.65 mM (Fig. 3). The AlaSal showed significantly less cytotoxicity against fibroblasts, even at higher concentrations: values up to about 30% of dead cells were noted at 7.30 mM [33]. PhAlaSal stored for two weeks diminished AGS cell viability when applied at 1.82–3.65 mM (55–75% of dead cells), with reduced toxicity (29% dead cells) noted for a concentration equal to 0.91 mM. Interestingly, despite its low inhibitory properties against L929 cells, AlaSal significantly diminished AGS viability for both the freshly-prepared and stored solution (35–70% of dead cells within the range of 0.91–7.30 mM) (Fig. 4). In turn, in the same concentration range, AlaSal had less effect on HeLa cell viability (24–49% of dead cells), and only freshly-prepared PhAlaSal significantly diminished the ability of HeLa cells to reduce MTT (38–47% of dead cells) within the range 0.91–3.65 mM (Fig. 3). Moreover, at any tested concentration, the AlaSal solutions did not induce nuclear rupture in the tested cells. (Fig. S9, [33]). PhAlaSal caused a weak tendency to cell nuclei vesication in 24%, 27% and 19% of the L929, AGS and HeLa cells, respectively, at 3.65 mM (Fig. S10).

Although PhAlaSal showed lower MIC values than AlaSal for more strains (Table 2, [33]), it demonstrated significant cytotoxic effects on L929 cells in the same concentration range, limiting its use as an antimicrobial drug. However, the results of inhibition of AGS and HeLa cells support its use against cancer cells. In contrast, AlaSal was found to be non- or weakly cytotoxic to fibroblasts and highly cytotoxic to cancer cells at concentrations that were bacteriostatic for the tested strains and bactericidal for *P. aeruginosa*. AlaSal may therefore be valuable in the treatment of *H. pylori* infections, which can lead to the development of gastric carcinoma, one of the leading causes of death from cancer [33], [68]. The ligand also showed fungistatic activity against *Candida* strains. The existence of *H. pylori* and *Candida* in the gastric mucosa synergistically affects the development of gastric diseases [69]. Therefore, it is very significant that AlaSal treatment resulted in high mortality among gastric adenocarcinoma epithelial cells in the MTT study: AlaSal treatment may serve as both antimicrobial therapy against *H. pylori* and *Candida* and antitumor therapy against AGS cells. Anticancer therapies based on PhAlaSal should also be developed in the future; although due to its high cytotoxicity against L929 and ligand-induced damage to cell nuclei, they may be less applicable than AlaSal-based therapies.

The Co(II)–PhAlaSal complexes showed a significantly cytotoxic effect towards L929 cells within the range 0.06–0.91 mM (freshly prepared; 32–84% of dead cells) and within the range 0.23–0.91 mM (stored for two weeks; 31–86% of dead cells) (Fig. 3). In the L929 cell cultures exposed to Co(II)–PhAlaSal complexes at a concentration of 0.46–0.91 mM (in both tested variants), approximately 31% of cells showed a tendency toward cell nucleus vesication (Fig. S10), as compared to unexposed control cells. In the case of AGS, the complexes in both solutions induced death in 16–46% cells (concentration range 0.23–0.91 mM) with the freshly-prepared compounds contributing to greater cell mortality. Of the AGS cells exposed to the freshly-prepared complexes only, 19% were degraded at 0.91 mM. The freshly-prepared solutions also exhibited a significantly greater cytotoxic effect against HeLa cells (37–77% of dead cells) than the solutions stored for two weeks (29–66% of dead cells at the concentrations 0.46–0.91 mM), even when applied at lower concentrations (0.11–0.91 mM). Co(II)–PhAlaSal complexes degraded the cell nucleus in approx. 40% of HeLa cells at 0.46–0.91 mM (freshly prepared).

In turn, the solution containing Co(II)–AlaSal complexes, stored for two weeks, has a stronger inhibitory effect towards L929 cells than the freshly-prepared material [33]. The same relationship is observed regarding cancer cells (Fig. 4), indicating that the complexes in the Co(II)–PhAlaSal or Co(II)–AlaSal systems have different modes of action on cell structures depending on their coordination geometry, viz. octahedral or tetrahedral. After storage for two weeks, 0.23–7.30 mM Co(II)–AlaSal solution significantly diminished the ability of AGS cells to reduce MTT (34–84% of dead cells) and demonstrated a cytotoxic effect toward HeLa cells (33–80% of dead cells). The presence of the complexes also caused slight damage to cell nuclei (up to 10%) of both types of cancer cells (Fig. S9).

The Co(II)–PhAlaSal and Co(II)–AlaSal formulations had adverse effects on L929 eukaryotic cells when used in the tested concentration range, suggesting that they may not be safe for antimicrobial applications. However, due to the cytotoxic effects of these compounds on AGS and HeLa cells, they can be used in further studies on the mechanisms of limiting the growth of cancer cells.

## 4 Conclusions

Potentiometric measurements followed by ESI–MS confirm the formation of four cobalt(II) complexes with the reduced Schiff base, *N*-(2-hydroxybenzyl)phenylalanine (PhAlaSal). A greater number of complexes is observed in the system with cobalt(II) than with other divalent transition metals. The overall stability constants of all the species were calculated. Coordination by amino, phenolic and carboxyl groups results in the formation of five- and six-membered chelate rings, increasing the stability of the complexes. Through HypSpec deconvolution, UV-Vis studies identified the octahedral [CoL_2_]^2-^ complex; moreover, the presence of the isosbestic point in a strongly alkaline environment, supported by the species distribution in the Co(II)–PhAlaSal system, indicates the appearance of a tri-ligand complex. A time-dependent change in absorbance was observed for the Co(II)–PhAlaSal system, during which the single- and bi-ligand forms changed structure from octahedral to tetrahedral.

Both the Co(II)–PhAlaSal species and the ligand alone, existing in an equilibrium mixture at physiological pH, exhibited antimicrobial activity. The complexes demonstrated the highest bacteriostatic and bactericidal efficacy against two strains of *H. pylori*; however, the ligand demonstrated lower antimicrobial activity towards *H. pylori* than the complexes. Despite this, PhAlaSal alone appears a more effective antimicrobial than the analogous Schiff base derivative (AlaSal) probably due to the presence of the benzyl group. Nevertheless, the cytotoxic effect of PhAlaSal on L929, AGS and HeLa cells with ligand-induced nuclear damage limits its application as an antimicrobial drug, but favors use against cancer cells. In contrast, AlaSal demonstrates very low cytotoxicity for fibroblasts and high cytotoxicity for cancer cells at concentrations inhibiting the growth of *H. pylori* and *Candida* strains; as such, AlaSal therapy may be a useful tool against both these microorganisms and AGS cells.

The complexes formed in the Co(II)–PhAlaSal and Co(II)–AlaSal systems exert a cytotoxic effect on both fibroblasts and cancer cells in the studied concentration range; however, the AlaSal-containing species are less likely to induce irreversible damage to cell nuclei. Although both types of complexes demonstrate significant cytotoxic activity, inhibiting the growth of cancer cells, they may not be suitable for treating fungal and bacterial infections.

Therefore, further experimental studies are needed to determine the potential of the individual complexes, identified at physiological pH in an equilibrium mixture, against adenocarcinoma of the cervix and gastric region.

## CRediT authorship contribution statement

**Magdalena Woźniczka**: Conceptualization, Methodology, Formal analysis, Investigation, Writing − original draft, Writing − review & editing, Visualization, Supervision, Project administration. **Mirosława Świątek**: Writing − review & editing. **Manas Sutradhar**: Resources, Writing − review & editing. **Joanna Gądek-Sobczyńska**: Writing − review & editing, Visualization. **Magdalena Chmiela**: Writing − review & editing. **Weronika Gonciarz**: Formal analysis, Investigation, Writing − original draft, Writing − review & editing. **Beata Pasternak**: Formal analysis, Investigation. **Marek Pająk**: Formal analysis, Investigation, Writing − review & editing.

## Declaration of Competing Interest

The authors declare that they have no known competing financial interests or personal relationships that could have appeared to influence the work reported in this paper.

## Appendix A Supplementary data

Species distribution curves (Fig. S1), mass spectra (Figs. S2-S6), electronic absorption spectra (Fig. S7, S8) and percentage of cells with damaged cell nuclei (Figs. S9, S10) can be found.
