# Supplementary material for: Equilibria of complexes in the aqueous cobalt(II)–N-(2-hydroxybenzyl)phenylalanine system and their biological activity compared to analogous Schiff base structures
Source: Comput Struct Biotechnol J. 2023 Jan 27;21:1312–23. doi: 10.1016/j.csbj.2023.01.035 (PMC9939546; doi:10.1016/j.csbj.2023.01.035)
Supplement: Supplementary file 1 — Supplementary material [file mmc1.doc]

Supplementary Information for the paper

**Equilibria of complexes in the aqueous**

**cobalt(II)–*N*-(2-hydroxybenzyl)phenylalanine system and their biological activity compared to analogous Schiff base structures**

Magdalena Woźniczka^a*^, Mirosława Świątek^a^, Manas Sutradhar^b,c^, Joanna Gądek-Sobczyńska^a^, Magdalena Chmiela^d^, Weronika Gonciarz^d^, Beata Pasternak^e^, Marek Pająk^a^

^a^ Department of Physical and Biocoordination Chemistry, Faculty of Pharmacy, Medical University of Lodz, Muszyńskiego 1, 90-151 Lodz, Poland

^b^ Faculdade de Engenharia, Universidade Lusófona de Humanidades e Tecnologias, Campo Grande 376, Lisboa1749-024, Portugal

^c^ Centro de Química Estrutural, Instituto Superior Técnico, Universidade de Lisboa, Av. Rovisco Pais, 1049-001 Lisboa, Portugal

^d^ Department of Immunology and Infectious Biology, Institute of Microbiology, Biotechnology and Immunology, Faculty of Biology and Environmental Protection, University of Lodz, Banacha 12/16, 90-237 Lodz, Poland

^e^ Department of Organic Chemistry, Faculty of Chemistry, University of Lodz, Tamka 12, 91-403 Lodz, Poland

^*^ Corresponding author. E-mail address: magdalena.wozniczka@umed.lodz.pl

Page 3. **Figure S1.** Species distribution curves as a function of pH for PhAlaSal ionic forms; *C*_PhAlaSal_ = 5×10^-3^ M.

Page 4. **Figure S2.** Tandem mass spectrum of the adduct [LH_2_ + NO_2_ + H]^+^ (*m/z* = 318.0), *C*_PhAlaSal_ = 5×10^-3^ M.

Page 5. **Figure S3.** ESI-MS spectra for ligand in positive ion-mode at various pH, *C*_PhAlaSal_ = 5×10^-3^ M.

Page 6. **Figure S4.** ESI-MS spectra for ligand in negative ion-mode at various pH, *C*_PhAlaSal_ = 5×10^-3^ M.

Page 7. **Figure S5.** Positive-ion ESI-MS spectra for the complexes formed in the Co(NO_3_)_2_/PhAlaSal system at ligand-to-metal molar ratio 2:1, at various pH, *C*_PhAlaSal_ = 5×10^-3^ M.

Page 8. **Figure S6.** Negative-ion ESI-MS spectra for the complexes formed in the Co(NO_3_)_2_/PhAlaSal system at ligand-to-metal molar ratio 2:1, at various pH, *C*_PhAlaSal_ = 5×10^-3^ M.

Page 9. **Figure S7.** **(a)** UV spectra of PhAlaSal within the pH 2.44–11.01, *C*_PhAlaSal_ = 2×10^-4^ M; **(b)** Molar absorption coefficients of ligand forms.

Page 10. **Figure S8. (a)** UV/Vis spectra of the Co(II)–PhAlaSal system at ligand-to-metal molar ratio 2:1; *C*_PhAlaSal_ = 2×10^-3^ M. **(b)** Molar absorption coefficients of the [CoL_2_]^2-^ complex.

Page 11. **Figure S9.** The percentage of AGS and HeLa cells with damaged cell nuclei. The cells were stimulated for 24 hours with: AlaSal or Co(II)–AlaSal complexes and then stained by 4’,6-diamidino-2-phenylindole (DAPI).

Page 12. **Figure S10.** The percentage of L929, AGS and HeLa cells with damaged cell nuclei. The cells were stimulated for 24 hours with PhAlaSal, Co(II)–PhAlaSal complexes or Co(II) alone and then stained by 4’,6-diamidino-2-phenylindole (DAPI). Statistical significance: *■p < 0.05; *untreated cells vs cells treated with tested solution (solution prepared directly); ■ untreated cells vs cells treated with tested solution (solution stored for two weeks).


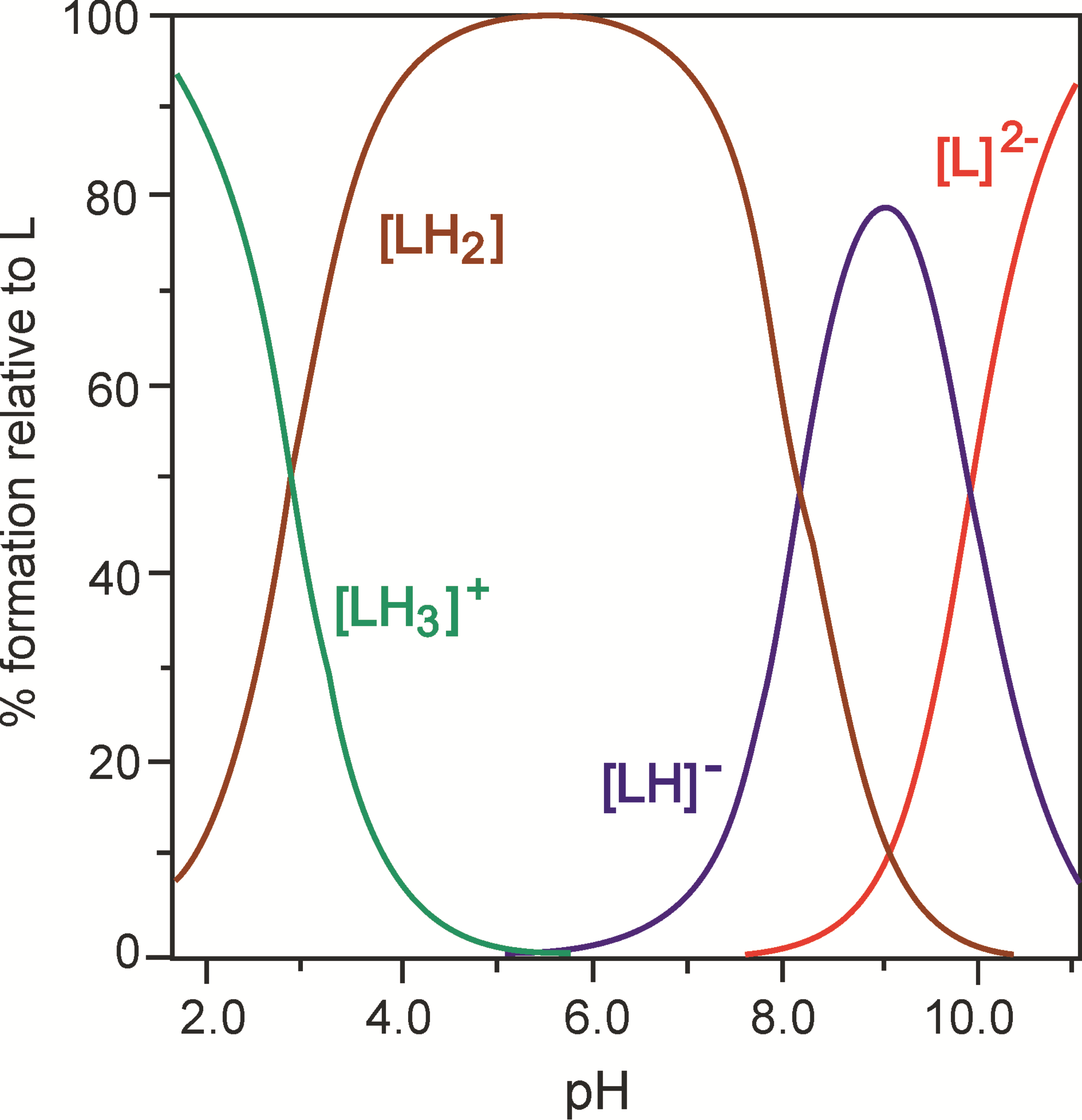


**Figure S1.** Species distribution curves as a function of pH for PhAlaSal ionic forms; *C*_PhAlaSal_ = 5×10^-3^ M.


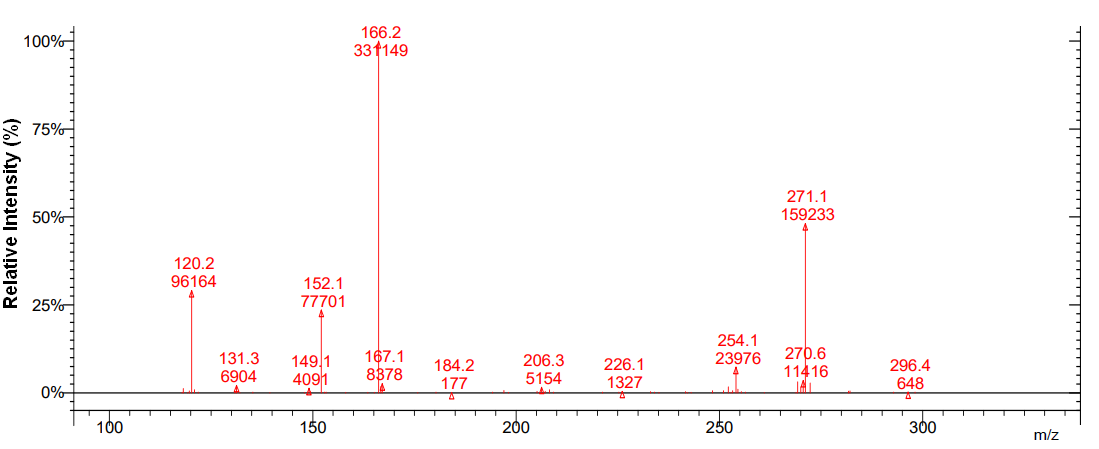


**Figure S2.** Tandem mass spectrum of the adduct [LH_2_ + NO_2_ + H]^+^ (*m/z* = 318.0), *C*_PhAlaSal_ = 5×10^-3^ M.


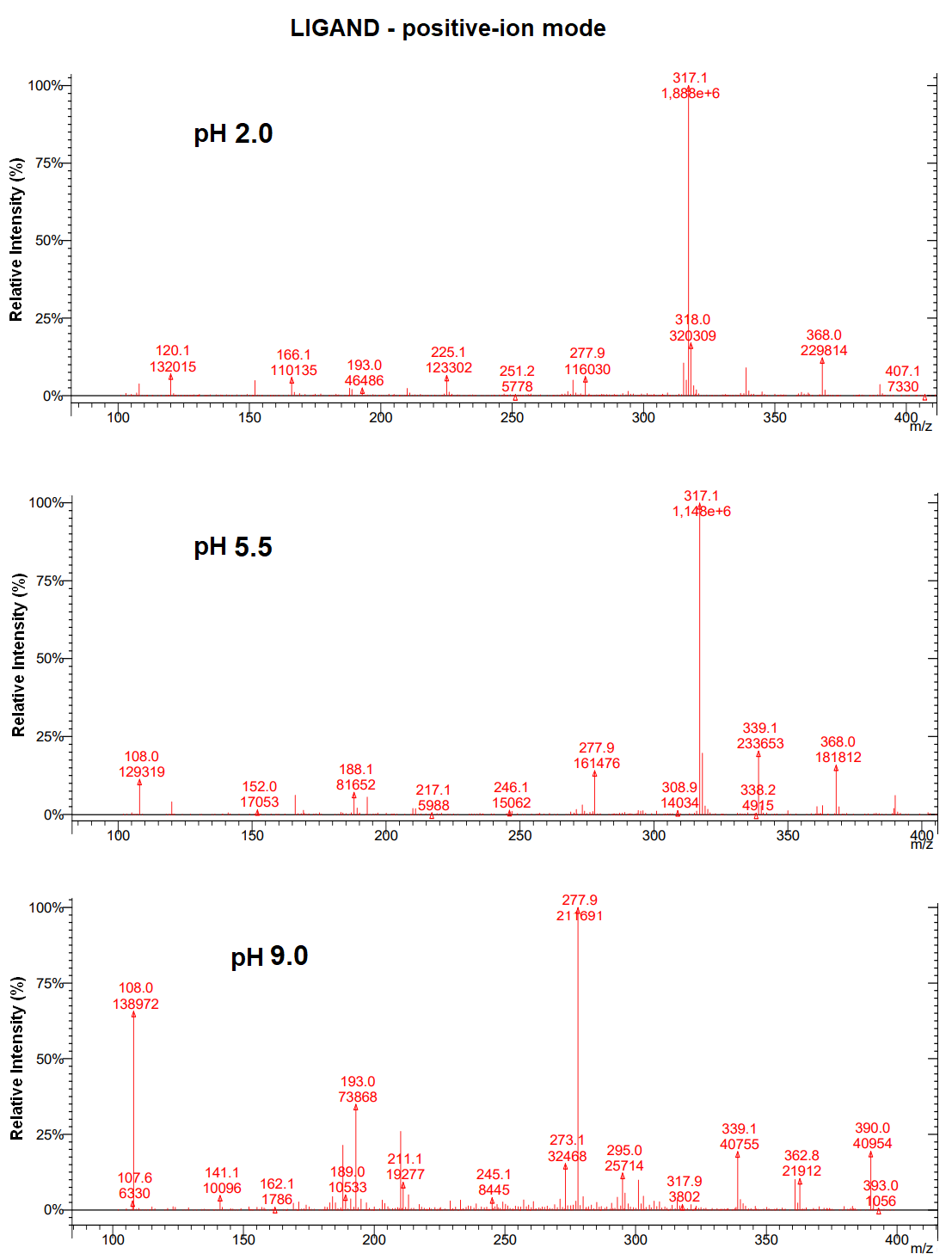


**Figure S3.** ESI-MS spectra for ligand in positive ion-mode at various pH, *C*_PhAlaSal_ = 5×10^-3^ M.


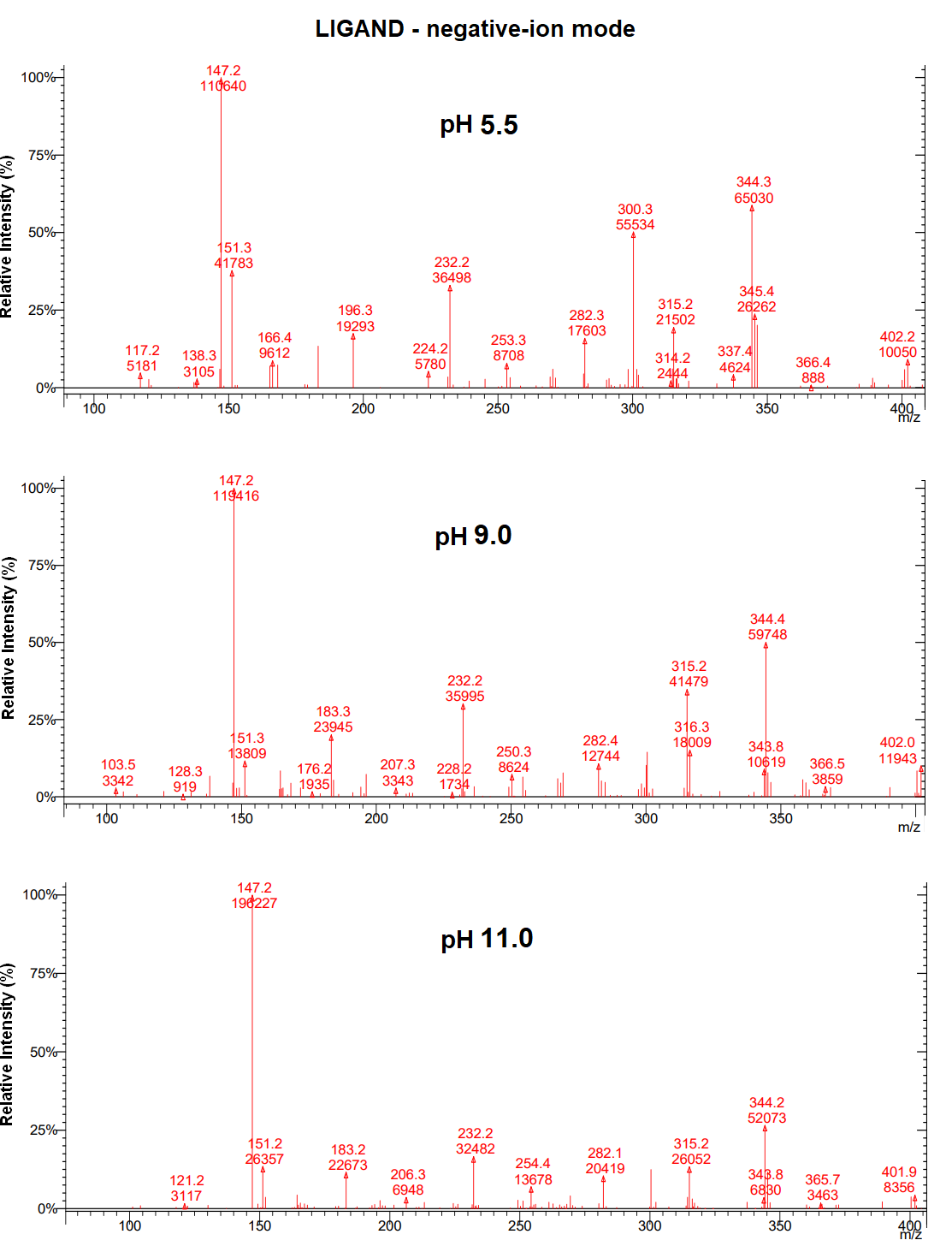


**Figure S4.** ESI-MS spectra for ligand in negative ion-mode at various pH, *C*_PhAlaSal_ = 5×10^-3^ M.


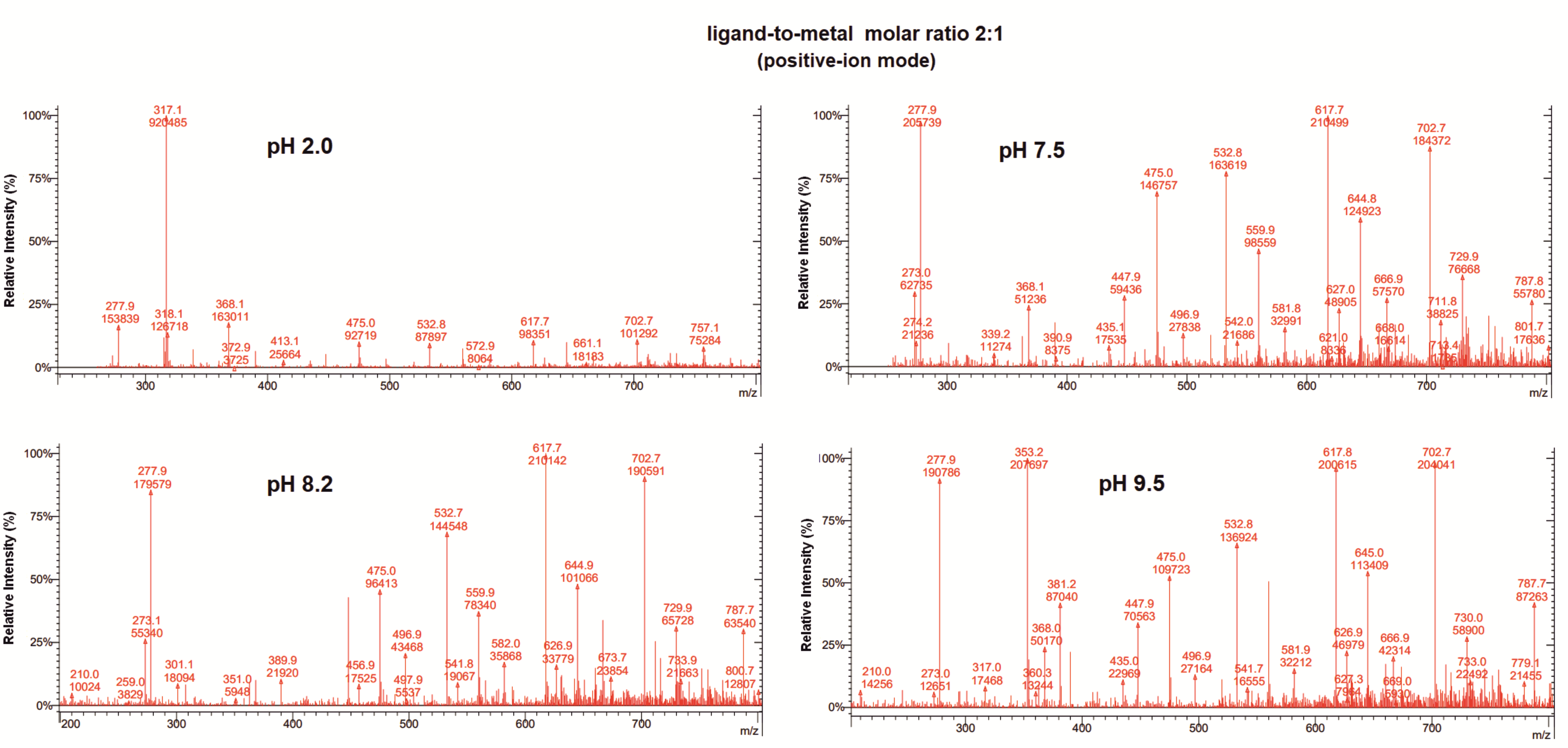


**Figure S5.** Positive-ion ESI-MS spectra for the complexes formed in the Co(NO_3_)_2_/PhAlaSal system at ligand-to-metal molar ratio 2:1, at various pH, *C*_PhAlaSal_ = 5×10^-3^ M.


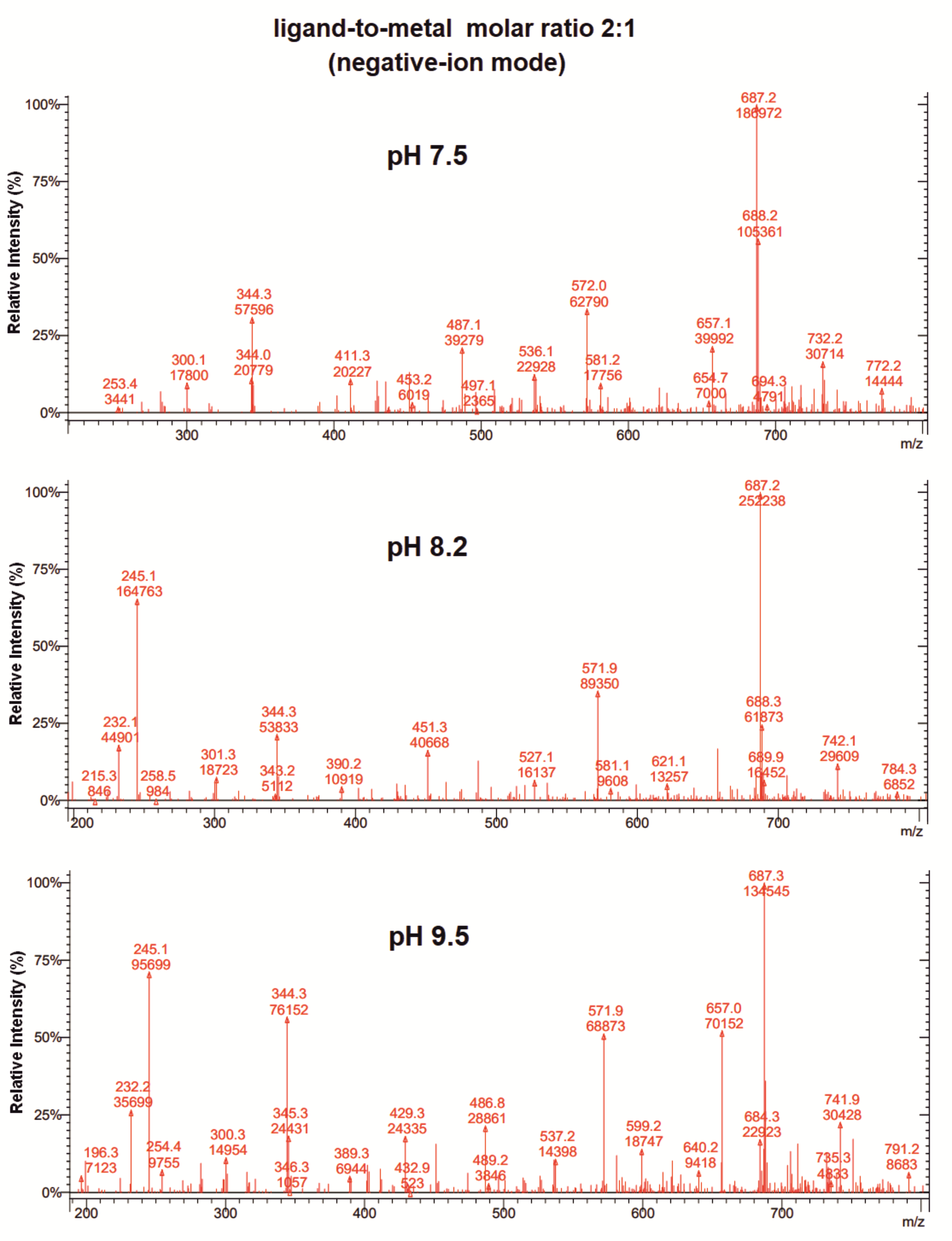


**Figure S6.** Negative-ion ESI-MS spectra for the complexes formed in the Co(NO_3_)_2_/PhAlaSal system at ligand-to-metal molar ratio 2:1, at various pH, *C*_PhAlaSal_ = 5×10^-3^ M.


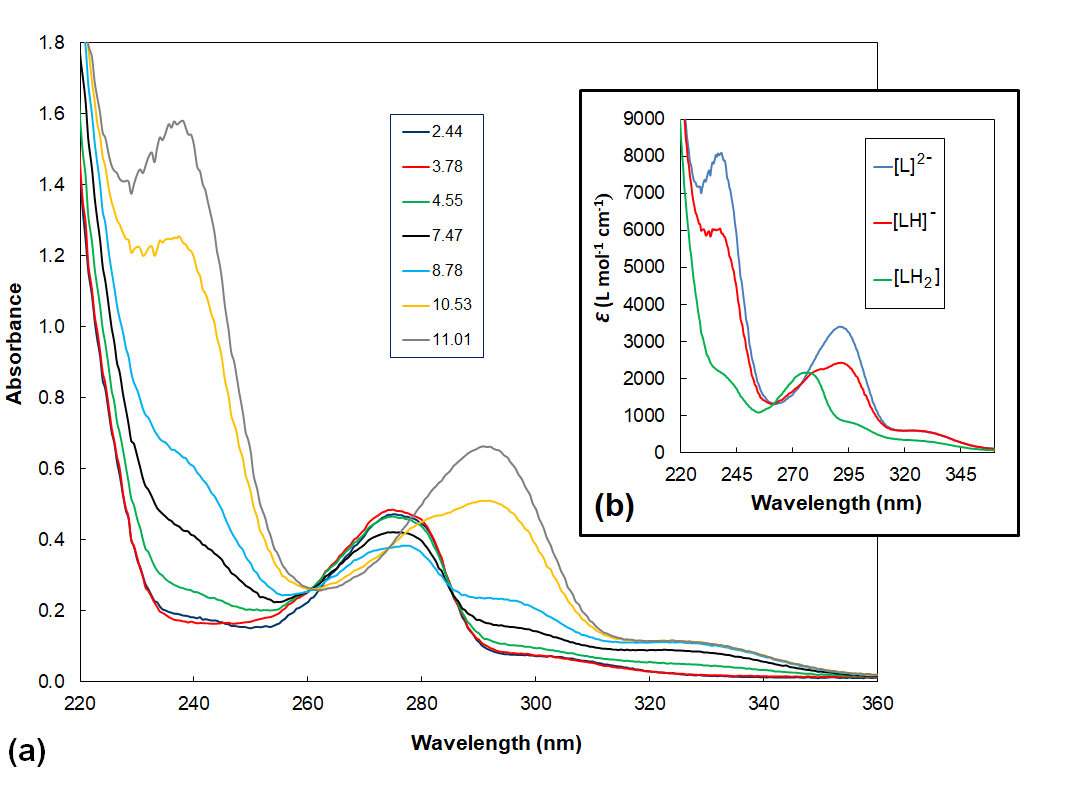


**Figure S7.** **(a)** UV spectra of PhAlaSal within the pH 2.44–11.01, *C*_PhAlaSal_ = 2×10^-4^ M;

**(b)** Molar absorption coefficients of ligand forms.


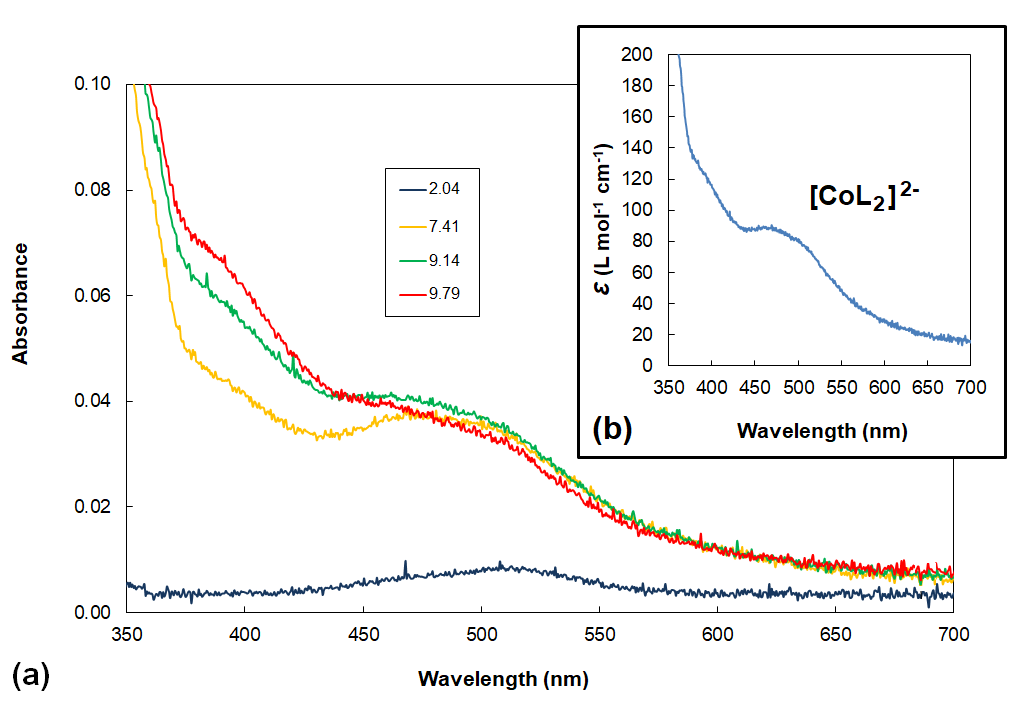


**Figure S8. (a)** UV/Vis spectra of the Co(II)–PhAlaSal system at ligand-to-metal molar ratio 2:1; *C*_PhAlaSal_ = 2×10^-3^ M. **(b)** Molar absorption coefficients of the [CoL_2_]^2-^ complex.


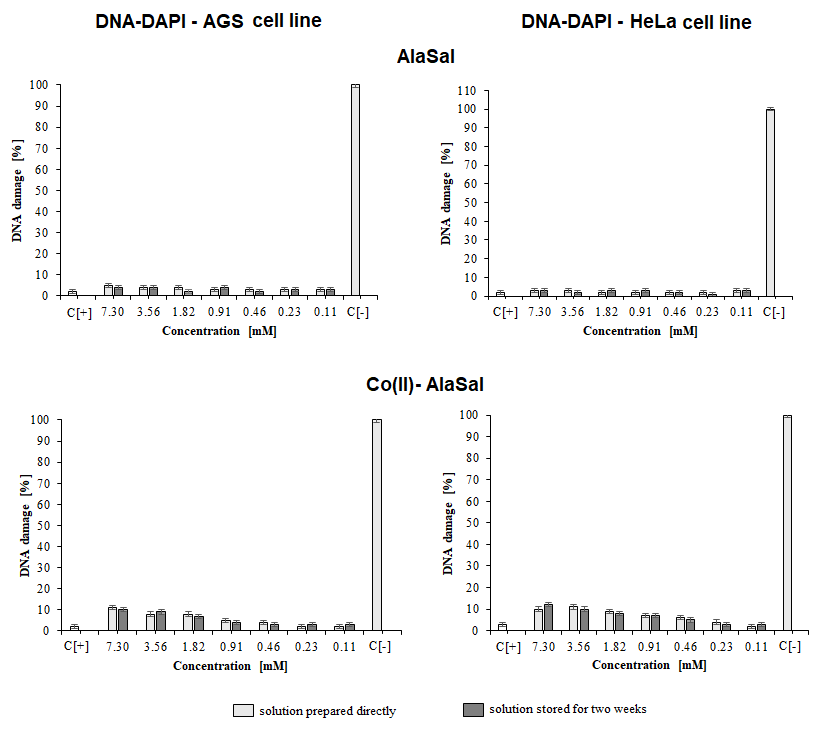


**Figure S9.** The percentage of AGS and HeLa cells with damaged cell nuclei. The cells were stimulated for 24 hours with: AlaSal or Co(II)–AlaSal complexes and then stained by 4’,6-diamidino-2-phenylindole (DAPI).


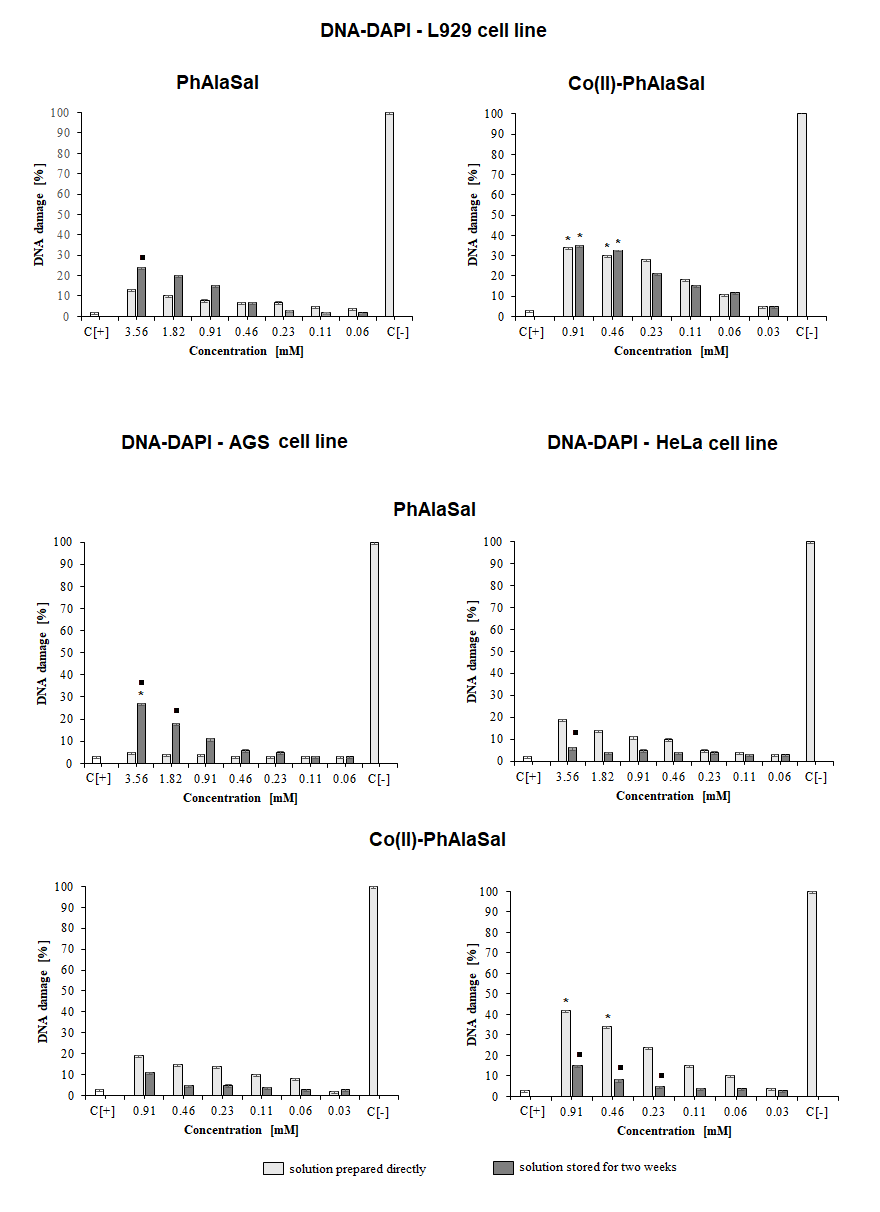


**Figure S10.** The percentage of L929, AGS and HeLa cells with damaged cell nuclei. The cells were stimulated for 24 hours with PhAlaSal, Co(II)–PhAlaSal complexes or Co(II) alone and then stained by 4’,6-diamidino-2-phenylindole (DAPI). Statistical significance: *■p < 0.05; *untreated cells vs cells treated with tested solution (solution prepared directly); ■ untreated cells vs cells treated with tested solution (solution stored for two weeks).
